# Meteorological influences on co-occurrence of O_3_ and PM_2.5_ pollution and implication for emission reductions in Beijing-Tianjin-Hebei

**DOI:** 10.1007/s11430-022-1070-y

**Published:** 2023-05-04

**Authors:** Xiaoqing Ma, Zhicong Yin, Bufan Cao, Huijun Wang

**Affiliations:** 1 Key Laboratory of Meteorological Disaster, Ministry of Education/Joint International Research Laboratory of Climate and Environment Change (ILCEC)/Collaborative Innovation Center on Forecast and Evaluation of Meteorological Disasters (CIC-FEMD), Nanjing University of Information Science & Technology, Nanjing, 210044 China; 2 Southern Marine Science and Engineering Guangdong Laboratory (Zhuhai), Zhuhai, 519080 China; 3 Nansen-Zhu International Research Centre, Institute of Atmospheric Physics, Chinese Academy of Sciences, Beijing, 100029 China

**Keywords:** Co-occurrence pollution, Ozone, PM_2.5_, Meteorology, Emission reduction

## Abstract

Co-occurrence of surface ozone (O_3_) and fine particulate matter (PM_2.5_) pollution (CP) was frequently observed in Beijing-Tianjin-Hebei (BTH). More than 50% of CP days occurred during April–May in BTH, and the CP days reached up to 11 in two months of 2018. The PM_2.5_ or O_3_ concentration associated with CP was lower than but close to that in O_3_ and PM_2.5_ pollution, indicating compound harms during CP days with double-high concentrations of PM_2.5_ and O_3_. CP days were significantly facilitated by joint effects of the Rossby wave train that consisted of two centers associated with the Scandinavia pattern and one center over North China as well as a hot, wet, and stagnant environmental condition in BTH. After 2018, the number of CP days decreased sharply while the meteorological conditions did not change significantly. Therefore, changes in meteorological conditions did not really contribute to the decline of CP days in 2019 and 2020. This implies that the reduction of PM_2.5_ emission has resulted in a reduction of CP days (about 11 days in 2019 and 2020). The differences in atmospheric conditions revealed here were helpful to forecast the types of air pollution on a daily to weekly time scale. The reduction in PM_2.5_ emission was the main driving factor behind the absence of CP days in 2020, but the control of surface O_3_ must be stricter and deeper.

**Electronic Supplementary Material:**

Supplementary material is available in the online version of this article at 10.1007/s11430-022-1070-y.

## 1. Introduction

Severe air pollution events frequently occurred in the east of China, especially in Beijing-Tianjin-Hebei (BTH) region, the Yangtze River Delta (YRD), and the Pearl River Delta. In recent years, the concentration of fine particulate matter ($PM_{2.5}$) has been decreasing across China due to the implementation of a variety of emissions control measures (Zhai et al., 2019). However, the concentration of $PM_{2.5}$ in BTH remained above the National Ambient Air Quality Standard in 2020 (Yang et al., 2022). At the same time, surface $O_3$ concentration has been increasing since 2013 in BTH (Wang et al., 2020a). Li et al. (2020) reported that the average daily maximum 8-hour concentration of ozone (MDA8 $O_3$) exceeded 160 μg $m^{-3}$ in most areas of eastern China in the summers from 2013 to 2019. In addition to uni-$O_3$ or $PM_{2.5}$ pollution, the co-occurrence of $O_3$ and $PM_{2.5}$ pollution (CP) were frequently observed in the east of China

(Zhang et al., 2022). Even during the COVID-19 lockdown period, simultaneous increases in $PM_{2.5}$ and $O_3$ concentrations were observed in January 2020 (Li et al., 2021). According to China National Ambient Air Quality Standard, a CP day was defined as a day when MDA8 $O_3$ was higher than 160 μg $m^{-3}$ and $PM_{2.5}$ was higher than 75 μg $m^{-3}$. CP days mainly appeared in the warm season in the BTH region, especially in April–May when more than 50% of CP days appear in the whole year (red bar in Figure 1a). The CP days greatly increased the complexity and difficulty of air pollution control in the east of China (Tie et al., 2019).

Exposure to $PM_{2.5}$ and $O_3$ can lead to a range of harmful effects on human health, but the health effects of these two pollutants are somewhat different. The $PM_{2.5}$-related mortality was dominated by cardiovascular mortality (Krewski et al., 2009), whereas $O_3$ was primarily associated with respiratory mortality (Jerrett et al., 2009). Co-occurring air pollution caused by $O_3$ and $PM_{2.5}$ can have compound harmful impacts and pose an overlay of potential health risks (Anenberg et al., 2010; Schnell and Prather, 2017). Thereby, a comprehensive understanding of the CP days is essential for the cooperative control of these two pollutants and for reducing additive disastrous effects.

The variabilities of $O_3$ and $PM_{2.5}$ were determined by joint effects of emissions and meteorological conditions. Ground-level $O_3$ was produced by a photochemical reaction of nitrogen oxides and volatile organic compounds under favorable meteorological conditions (Xue et al., 2014). In addition to direct emissions of primary aerosols (such as black carbon and mineral dust), $PM_{2.5}$ can also come from chemical transformations of gaseous precursors such as nitrogen oxides, sulfur dioxide, and ammonia in the atmosphere (Wu et al., 2021). The precursors of $PM_{2.5}$ and $O_3$ were not exactly the same (Yin et al., 2021). Moreover, more than 70% of the observed daily variation in summer $O_3$ concentration can be explained by daily variations in key meteorological parameters (Qian et al., 2022). Therefore, the variation in anthropogenic emission did not have a great impact on the simultaneous changes in $PM_{2.5}$ and $O_3$ concentrations on the daily time scale. The anthropogenic emission mainly affected the trend of air pollution. For example, the persistent decrease in $PM_{2.5}$ is a result of the intensified air pollution management that has been implemented since 2013 (Wang et al., 2020b).

Meteorological conditions played an important role in the co-occurrence of $O_3$ and $PM_{2.5}$ pollution. Changes in the meteorological condition in different regions have resulted in significant regional differences in CP day in the east of China (Zong et al., 2021). In the YRD, the total number of CP days decreased during 2015–2019 (Qin et al., 2021). Two-thirds of the CP days in the YRD occurred in late spring and early summer, which were related to stable and warm conditions under the influence of high-pressure systems (Dai et al., 2021). For the BTH region, the independent impacts of meteorological conditions on $PM_{2.5}$ or $O_3$ have been clearly illustrated in many previous studies (Dong et al., 2020; Wang et al., 2020b). The $PM_{2.5}$ concentration increase in BTH was often related to stagnant weather conditions and high relative humidity (Zhang et al., 2021), which was mainly induced by anomalous anticyclones that occurred in the middle troposphere above northeastern Asia (Zhong et al., 2019). The anomalous anticyclones over BTH were always accompanied by clear sky and high temperatures in summer, both of which were favorable for the increase in $O_3$ concentration and thereby were conductive to $O_3$ pollution (Cao and Yin, 2020). It is important to note that local atmospheric anomaly in BTH associated with $O_3$ and $PM_{2.5}$ pollution are similar, i.e., an anticyclone always occur in the middle troposphere. However, to the best of our knowledge, the meteorological impact on CP days in BTH has not been comprehensively described yet and the likely impact of emission reduction taken in recent years on CP days also remains unknown. In this study, the features of $O_3$ and $PM_{2.5}$ co-pollution and meteorological impacts during April–May were analyzed. Furthermore, the potential impact of emission reduction on CP days was also discussed. The findings are expected to provide scientific support for the forecast and evaluation of air pollution in BTH.

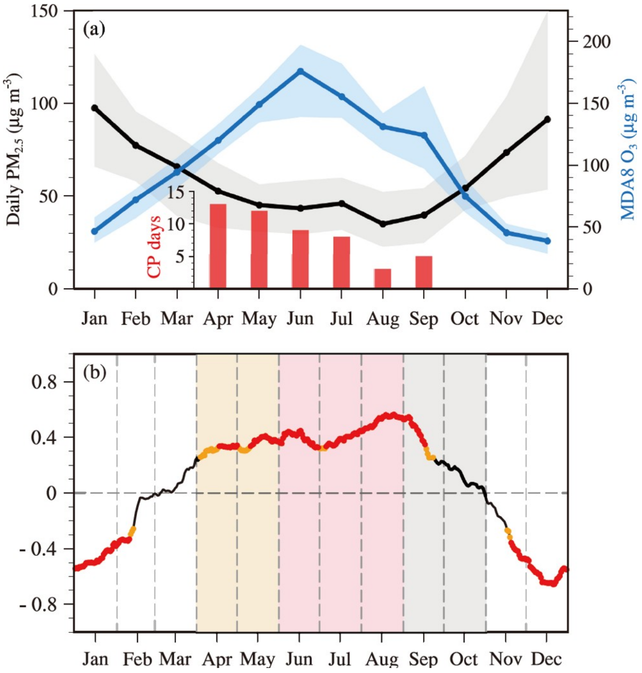

**Figure 1** (a) Variations in monthly mean $PM_{2.5}$ concentration (unit: μg $m^{-3}$, black), MDA8 $O_3$ concentration (unit: μg $m^{-3}$, blue), and the total number of CP days (unit: days, red bar) during 2015–2020. Shadings represent the range of pollutant concentrations from 2015 to 2020. (b) Mean of 60-day sliding correlation coefficients between MDA8 $O_3$ and daily $PM_{2.5}$ in BTH during 2015–2020. The red (orange) dots indicate that the correlation coefficients are significantly above the 99% (95%) confidence level. The vertical lines separate the months.

## 2. Datasets and methods

Hourly observations of $PM_{2.5}$ and $O_3$ concentrations from 2015 to 2020 are publicly available at https://quotsoft.net/air/ (last access: 4 March 2022). The stations with more than 5% of the data missed were eliminated, and observations at 67 stations in BTH were employed in this study. The average concentration of 67 sites was used as the pollutant concentration in BTH. Various meteorological variables with the horizonal resolution of 0.125°×0.125° for the period 2015–2020 are extracted from the Fifth Generation European Center for Medium-Range Weather Forecasts Reanalysis Dataset (ERA5, Hersbach et al., 2020). These variables include geopotential height at 500 hPa (Z500), zonal and meridional winds at 850 hPa, specific humidity at 925 hPa, surface relative humidity (RH), boundary layer height, downward solar radiation at the surface (Ssr), surface air temperature (SAT) and 10-m meridional and zonal winds (UV10).

According to China National Ambient Air Quality Standard, the concentration of MDA8 $O_3$ or daily $PM_{2.5}$ is above the Grade II air quality standard if MDA8 $O_3$ is higher than 160 μg $m^{-3}$ or daily $PM_{2.5}$ is higher than 75 μg $m^{-3}$. $PM_{2.5}$ pollution (PP) days are identified when the regional average (the average at 67 sites) of daily $PM_{2.5}$ concentration is above 75 μg $m^{-3}$ and $O_3$ pollution (OP) days are identified when the regional average of MDA8 $O_3$ is above 160 μg $m^{-3}$ in BTH (Figure 2a). During the CP days in BTH, area-mean concentrations of both MDA8 $O_3$ and daily $PM_{2.5}$ are above the Grade II standards; during none-pollution (NP) days, $O_3$ and $PM_{2.5}$ concentration is less than 100 and 35 μg $m^{-3}$, respectively (Grade I air quality standard, Figure 2a). In order to distinguish the difference between co-occurrence and uni-occurrence of $O_3$ and $PM_{2.5}$ pollution days, the CP days have been removed from PP and OP days.

To confirm the regional CP days defined in the present study, the percentage number of stations that experienced the CP day when a regional CP day occurred in BTH was calculated in April–May. We analyze 25 CP days that occurred in BTH during April–May of 2015–2020. In 92% of these CP days, over 50% of stations in BTH experienced co-pollution (Appendix Figure S1, https://link.springer.com). For a specific region, when air pollution occurs at more than 50% of the observation stations, the day can be defined as an air pollution day (Chen and Wang, 2015). When the regional pollution days were defined as occurring when the values of more than 50% of stations in BTH exceeded the aforementioned thresholds, and similar conclusions were obtained as in this paper (figure not shown). It is reasonable to identify a regional pollution day when the averages of $O_3$ and $PM_{2.5}$ concentrations at all 67 stations in BTH exceed the aforementioned thresholds.

## 3. Features of co-occurrence of $O_3$ and $PM_{2.5}$ pollution

The 60-day sliding correlation coefficients between $PM_{2.5}$ and $O_3$ from 2015 to 2020 were examined (Figure 1b). The relationship between $PM_{2.5}$ and $O_3$ displayed a large seasonality. $PM_{2.5}$ and $O_3$ concentrations showed a significant positive correlation in summer, whereas a significant negative correlation was found in winter (both passing the confidence level of 99%). Two transitional periods of the relationship were found. In September and October, the correlation between $PM_{2.5}$ and $O_3$ dramatically declined from a significant positive correlation to no correlation (gray shading in Figure 1b). Although the correlation between these two pollutants in April and May was lower than that in summer, it still presented a significant positive correlation

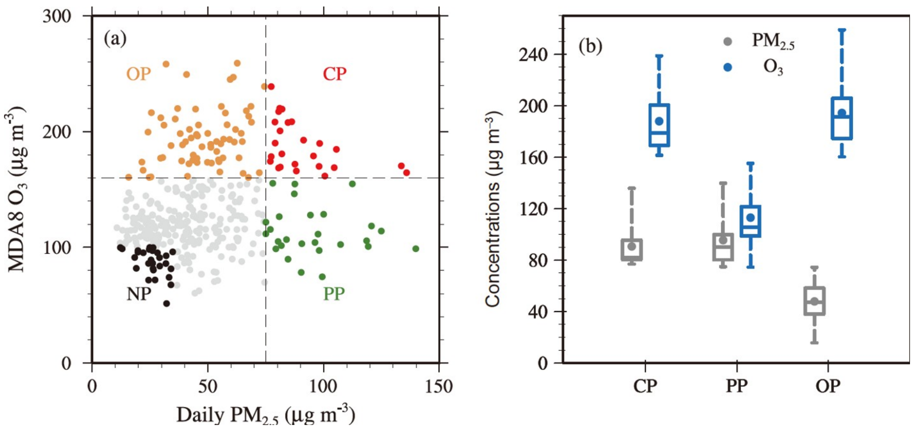

**Figure 2** (a) Scatter plots of daily $PM_{2.5}$ and MDA8 $O_3$ concentrations (unit: μg $m^{-3}$) in April and May during 2015–2020. CP, OP, PP, and NP days were denoted by red, orange, green, and black, respectively. The horizontal and vertical dashed line indicates the Grade II air quality standard of MDA8 $O_3$ (160 μg $m^{-3}$) and $PM_{2.5}$ (75 μg $m^{-3}$) values. (b) Boxplots of daily $PM_{2.5}$ (gray) and MDA8 $O_3$ (blue) concentrations (unit: μg $m^{-3}$) during CP, PP, and OP in April and May over BTH. The boxes enclose the 25th, 50th, and 75th percentiles, and the whiskers represent the values from minimum to maximum. The circles represent mean values.

(passing the confidence level of 95%, orange shading in Figure 1b).

High concentration of MDA8 $O_3$ mainly occurred from April to September, and the $O_3$ concentration decreased rapidly in the cold season (less than 100 μg $m^{-3}$, Figure 1a). On the contrary, $PM_{2.5}$ concentration often exhibited higher values from November to February and lower values in the warm season. Therefore, the co-occurrence of $PM_{2.5}$ and $O_3$ pollution rarely happened in winter and summer. It was worth noting that $PM_{2.5}$ concentration in April and May (48.7 μg $m^{-3}$) was a little higher than that in September and October (46.8 μg $m^{-3}$, Figure 1a). The positive correlation between $PM_{2.5}$ and $O_3$ in April and May and the relatively high concentrations of $PM_{2.5}$ and $O_3$ during this period resulted in the largest number of CP days in April and May (50% of annual total CP days, red bars in Figure 1a). Therefore, this study focused on $PM_{2.5}$ and $O_3$ co-polluted days in April and May and compared them with the $PM_{2.5}$ and $O_3$ pollution days.

According to the definitions of different types of pollution, $PM_{2.5}$ concentrations during PP days were significantly higher than that during OP days. Similarly, the concentrations of MDA8 $O_3$ during the OP days were also higher than that during the PP days. Specifically, the highest concentrations of MDA8 $O_3$ (259.1 μg $m^{-3}$) and $PM_{2.5}$ (139.8 μg $m^{-3}$) were both observed in OP and PP days. The mean concentrations of MDA8 $O_3$ and $PM_{2.5}$ during the OP (PP) were 194.5 and 48.0 (113.1 and 95.6) μg $m^{-3}$, respectively. The concentrations of MDA8 $O_3$ and $PM_{2.5}$ during the CP were 188.1 and 90.7 μg $m^{-3}$ respectively from 2015 to 2020 (Figure 2b). That is to say, the CP $PM_{2.5}$ was lower than but close to that in PP, and the CP $O_3$ was also lower than but close to that in OP. This result indicated that the pollution level of CP was high for $PM_{2.5}$ and $O_3$ and thus was much higher than the OP and PP days.

The number of CP days reached its highest value (11 days) in April and May of 2018, i.e., 11 days accounting for 44% of the total CP days. The mean number of CP days was 4 during 2015–2020, while it was 6 during the years of 2015–2018 and was only 2 in 2019 and 0 in 2020 (Figure 3a). Differently, the April–May number of OP days increased during 2015–2020 and exceeded 16 days in 2019 and 2020. The $O_3$ concentration also showed an increasing trend (3.7 μg $m^{-3}$ $yr^{-1}$) since 2015 (Figure 3b). On the contrary, the number of PP days significantly decreased and there were no $PM_{2.5}$ pollution days in April and May of 2020. Although the $PM_{2.5}$ concentration decreased by the rate of 5.6 μg $m^{-3}$ $yr^{-1}$ over 2015–2020, it was still at high levels before 2018 (the mean $PM_{2.5}$ concentration in 2018 was 50.6 μg $m^{-3}$, Figure 3b). It is possible that the enhancement of $O_3$ pollution, superimposed on high levels of particulate matter, prompts a large number of CP days from 2015 to 2018. However, the low $PM_{2.5}$ concentration is the critical reason for the disappearance of CP days in 2020.

## 4. Associated meteorological conditions

As well known, $PM_{2.5}$ and $O_3$ pollution was significantly influenced by meteorological conditions on a daily time scale (Fu et al., 2020; Yin and Ma, 2020). However, the impacts of meteorological conditions on CP days were still unclear, especially in BTH. In this section, we analyzed the anomalous atmospheric circulations associated with CP days by composite analysis and comparison with that during OP and PP days. The differences in meteorological condition were calculated with respect to NP days that were characterized by sufficient rainfall, cool air temperature, and anomalous northerly wind (figure not shown). Composite analyses were carried out for 2015–2020 and 2015–2019 (Figures 4, 5) respectively and identical results were obtained. Considering the absence of CP in 2020, the physical mechanisms were illustrated based on observations for the period from 2015 to 2019.

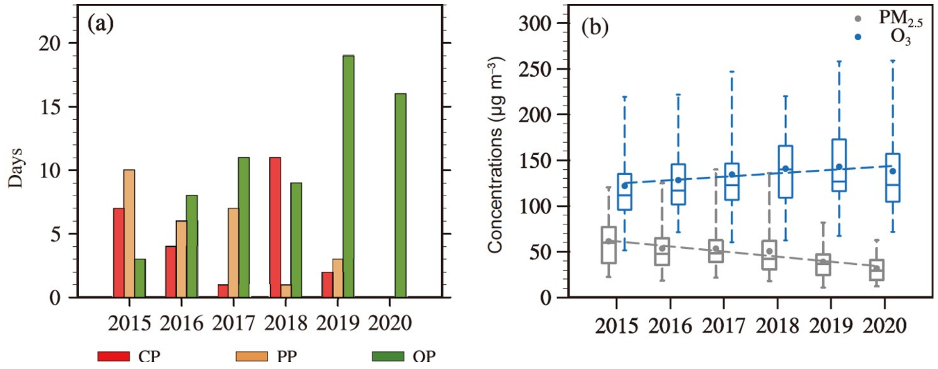

**Figure 3** (a) Numbers of CP (red), PP (orange), and OP (green) days in BTH in April–May of 2015–2020. (b) Boxplots of variations in April–May of $PM_{2.5}$ (gray) and MDA8 $O_3$ (blue) concentrations (unit: μg $m^{-3}$) during 2015–2020. The boxes enclose the 25th, 50th, and 75th percentiles and the whiskers represent the minimum to maximum values. The circles represent the mean values. The gray (blue) dashed line shows the linear trend of $PM_{2.5}$ (MDA8 $O_3$) from 2015 to 2020.

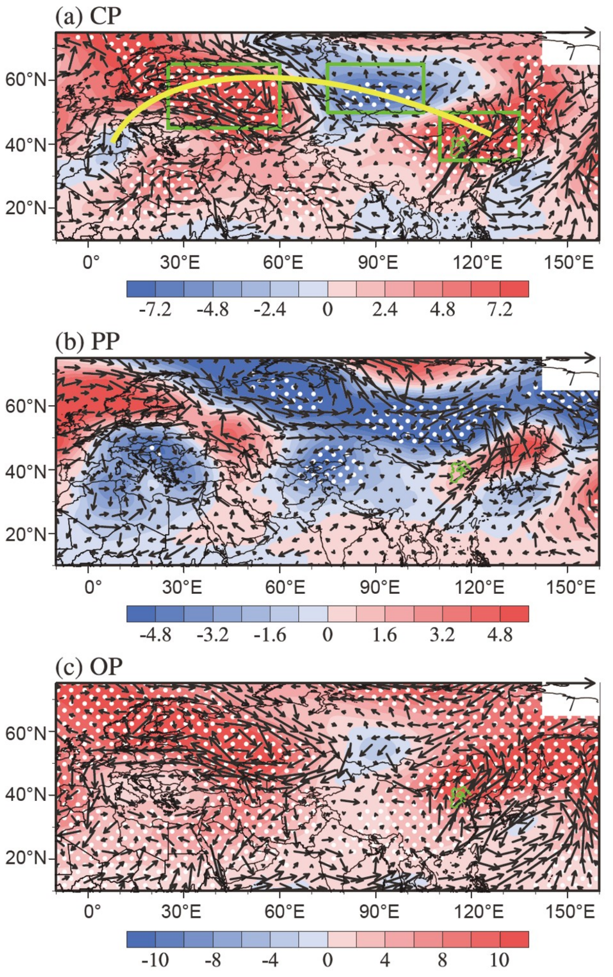

**Figure 4** Composites of Z500 (unit: 10 gpm, contours) and wind at 850 hPa (unit: m $s^{-1}$, arrows) associated with CP (a) , PP (b), and OP (c) days in April–May during 2015–2019 with respect to NP days. The white dots indicate that the differences are above the 95% confidence level. The yellow line shows the large circle path of the Rossby wave-like train. The green boxes in (a) represent the centers to calculate the SN index. The Beijing-Tianjin-Hebei area is also shown.

The Scandinavia pattern is a prominent teleconnection pattern that affects Eurasia's climate (Barnston and Livezey, 1987). It has three anomalous centers located over West Europe, west Russia, and Mongolia, respectively. In the positive phase of the Scandinavia pattern, blocking anticyclones over Scandinavia and the Ural Mountains likely intensify (Wang and Tan, 2020). As shown in Figure 4a, anticyclonic and cyclonic anomalies were alternately distributed over west Europe (−), Scandinavia, and the Ural Mountains (+) and to north Mongolia (−) during the positive phase of the Scandinavia pattern. In addition, another anomalous anticyclonic circulation can be observed over North China in the lower to middle troposphere. Because the atmospheric active center over west Europe was relatively weak, we defined a Rossby wave-like train that has two significant centers corresponding to the Scandinavia pattern and one center over North China (SN pattern) to further demonstrate atmospheric impacts on CP days. The SN index was calculated by the Z500 differences between the two positive centers and the negative center. Corresponding to the occurrence of a significant positive phase of SN pattern was observed two days, one day and 0 day in advance (i.e., SN index > its one standard deviation), 68%, 68% and 72% of CP days could be successfully captured. Such a large percentage indicated that the strengthening positive phase of SN pattern to a great extent contributed to the co-occurrence of $PM_{2.5}$ and $O_3$ pollution in BTH, and thus it could serve as a useful signal for the forecast of CP days.

The anomalous anticyclonic circulation over North and Northeast China, when with appropriate intensity and backward-tilting structure with altitude, was the critical and local system that resulted in CP days (Figure 4a). The anticyclonic anomaly in the upper troposphere implied descending motion and a cloudless sky, which frequently led to strong solar radiation and high SAT in BTH. In the middle troposphere at 500 hPa, the anticyclonic anomaly extended to the southeast. Pollutant precursors and abundant water vapor were transported from the Yangtze River delta and adjacent sea to North China by the southerly winds associated with the anticyclonic anomaly (Figure 5a, 5b). High temperatures and strong solar radiation were favorable for the natural emission of $O_3$ precursors and photochemical reactions in the atmosphere (Zhao et al., 2021). At the same time, the moist environment enhanced the hygroscopic growth of $PM_{2.5}$ components, while the capability of clean air from the north to disperse the accumulated pollutant particles was quite limited. As a result, the meteorological condition (i.e., hot, wet, and stagnant) modulated by such large-scale atmospheric anomalies became favorable for higher concentrations of $PM_{2.5}$ and $O_3$ that could exceed the Grade II air quality standard.

The SN pattern could not be clearly detected during both PP or OP days. During PP days, negative Z500 anomalies were located zonally from the Ural Mountains to Lake Baikal, which induced northerly winds over BTH area (Figure 4b). Meanwhile, the weak and narrow positive Z500 anomalies over Northeast China led to weak southerlies across the BTH area. Convergence of air flow in the middle and lower troposphere could promote the formation of clouds and thus reduce solar radiation. This explained why solar radiation on average decreases by 8.6 $10^5$ J $m^{-2}$ and was lower than $10.0\times10^5$ J $m^{-2}$ during CP days (Table 1). Consequently, SAT (14.6°C) was much lower and dramatically suppressed the formation of natural precursors and photochemical production of surface $O_3$. However, the southerly winds effectively brought moist air to the BTH area, which subsequently led to high relative humidity (50.6%) and strong hygroscopic growth of $PM_{2.5}$. In addition, the stagnant air greatly weakened the dispersion of $PM_{2.5}$ in BTH (Figure 5c, 5d). In April and May, large-scale atmospheric anomalies

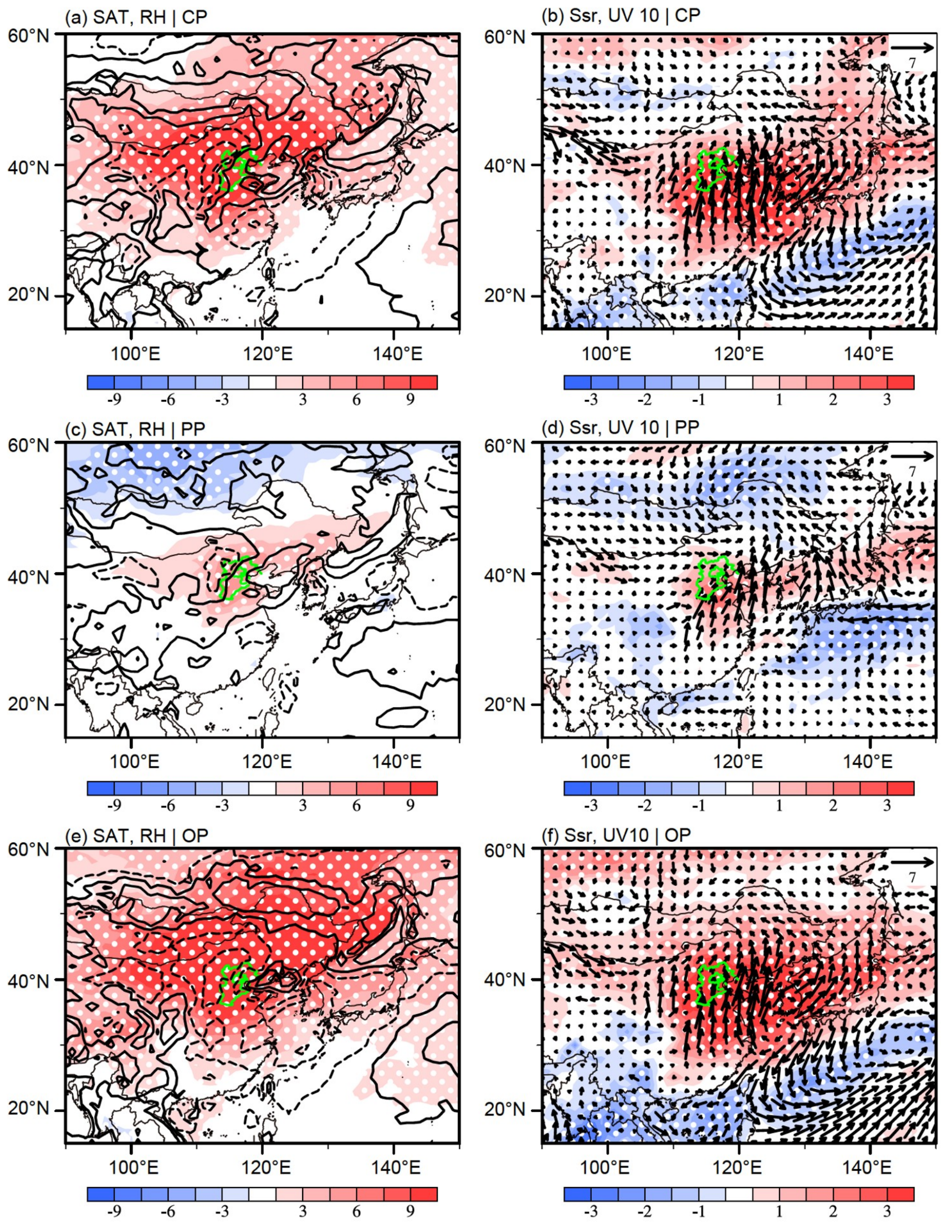

**Figure 5** Composites of meteorological conditions associated with ((a), (b)) CP, ((c), (d)) PP, and ((e), (f)) OP days in April–May during 2015–2019 with respect to NP days. The variables include ((a), (c), (e)) surface air temperature (SAT, unit: °C, shadings) and surface relative humidity (RH, unit: %, contours), ((b), (d), (f)) downward solar radiation at the surface (Ssr, unit: $10^5$ J m$^{-2}$, shadings) and surface winds (unit: m s$^{-1}$, arrows). The white dots indicate that the differences are above the 95% confidence level. The Beijing-Tianjin-Hebei area is also shown.

**Table 1** Area-averaged and standard error meteorological factors during CP, PP, and OP days in BTH from 2015 to 2019[a)]

| Variable name (unit) | CP | PP | OP |
|---|---|---|---|
| SAT (°C) | 20.8±0.7 | 14.6±0.7 | 21.5±0.4 |
| RH (%) | 49.7±1.7 | 50.6±2.3 | 37.3±1.4 |
| Ssr ($10^5$ J m$^{-2}$) | 10.0±0.3 | 8.6±0.4 | 10.4±0.2 |
| V10 (m s$^{-1}$) | 2.2±0.2 | 1.6±0.2 | 1.8±0.2 |

a) SAT, RH, Ssr, and V10 were surface air temperature, relative humidity at surface, downward solar radiation at the surface, and 10-m meridional winds, respectively.

as shown in Figure 4b, and associated meteorological conditions (Figure 5c, 5d) accelerated the accumulation of $PM_{2.5}$ particles on the one hand, but they also weaken the $O_3$ production in the atmosphere on the other hand.

Positive Z500 anomalies were vastly distributed over Eurasia and particularly strong anticyclonic anomalies prevailed over the east of China during OP days. It is important that an anomalous cyclonic circulation existed over the East China Sea at 850 hPa (Figure 4c). Such a configuration of anticyclonic and cyclonic anomalies could result in the divergence of water vapor in offshore area of China and thus little moisture could be transported from south to North China (Figure 5e, 5f). Note that relative humidity was only 37.3% during OP, which was lower than that during CP by 12.4% (Table 1). However, the inland southerly winds could still transport $O_3$ precursors from the Yangtze River Delta, where anthropogenic emissions were high due to the developed economy. The strong anticyclonic circulation anomalies induced abnormal descending motions and intense solar radiation. The SAT in BTH was 21.5°C on average during CP days, which was higher than that during PP days by 6.9°C. Such a high temperature and intense solar radiation were greatly favorable for the natural emission of $O_3$ precursors and photochemical reactions in the atmosphere. These large-scale atmospheric anomalies and meteorological conditions discussed above could greatly elevate $O_3$ concentration near the surface but reduce $PM_{2.5}$ particles in the BTH.

## 5. Implications of emission reduction

Daily variation in meteorological conditions significantly influenced the occurrence of $PM_{2.5}$ and $O_3$ co-polluted days. Meanwhile, the associated large-scale atmospheric anomalies also presented prominent differences from those affecting PP and OP days. Multiple linear regression (MLR) has been widely used to investigate the comprehensive effect of meteorological conditions on air pollutants (Zhai et al., 2019). The stepwise MLR model was trained once a year from 2015 to 2020 using meteorological variables associated with the SN pattern, and the daily $PM_{2.5}$ and $O_3$ concentrations then could be well reproduced by the MLR model. The correlation coefficients between MLR model outputs and observations can be above 0.5 and 0.8 for daily $PM_{2.5}$ and $O_3$ respectively (Figure S2, exceeding the 99.9% confidence level), which indicated that the impacts of meteorological conditions were approximate in April–May. The related meteorological conditions in Table 1 did not demonstrate obvious trend changes from 2015 to 2020 (figure not shown). The MLR model can well reproduce the means of pollutant concentrations and their trends only because the emission baseline of each year is implicitly expressed by the MLR coefficients (Yin et al., 2021). Particularly, surface relative humidity and meridional winds, which mainly influenced $PM_{2.5}$, did not show large differences in 2019 and 2020 compared to those during 2015–2018. Thus, the variation in April–May means the meteorological condition cannot explain the abrupt reduction of CP days since 2019. As displayed in Figure 3b, the April–May mean $O_3$ concentration has been increasing after 2015 and remains high in 2019 and 2020, which makes it impossible to reduce the CP days. The enhanced $O_3$ pollution was attributed to mismatched changes in volatile organic compounds and nitrogen oxides (Lu et al., 2019), and changes in hydroperoxyl radicals and solar radiation accompanied by decreased $PM_{2.5}$ (Zhu et al., 2019). Reasonably, the meteorological conditions cannot explain the disappearance of CP days, which implies that the persistent decrease in $PM_{2.5}$ concentration driven by emission reduction is probably the critical factor responsible for the disappearance of CP days.

To verify the above speculation, a fixed MLR model to produce $PM_{2.5}$ concentration was trained by the data in 2015. As explained by Yin et al. (2021), the emission baseline of 2015 was implicitly expressed by the coefficients of meteorological factors. When simulating the $PM_{2.5}$ concentration by daily meteorology from 2016 to 2020 and the fixed MLR model, the daily $PM_{2.5}$ concentration was calculated with the assumption of no emission reduction. In addition, the relationship between daily meteorology and $PM_{2.5}$ was well described in the fixed MLR model (Figure S2). Combining the simulated $PM_{2.5}$ and observed $O_3$, CP days could be detected when there was no impact of emission reduction on $PM_{2.5}$ pollution. The simulated CP days in 2015 occurred almost on the same days as observations (Figure 6a). Over time, almost all of the observed CP days could be detected based on the MLR model simulation, but the simulated number of CP days was larger than that of observations, indicating the obvious contribution of emission reduction on decreasing CP (Figure 7). To further enhance the robustness and reasonability, the mean bias correction (MBC) method was also employed in the present study. The MBC method was frequently used to improve the mean values predicted by numerical models (Kim et al., 2020). The intensity of anthropogenic emission largely determined the level of air pollution (Jeong et al., 2021), that is, the April–May mean of $PM_{2.5}$ concentration in each year. If we assume the $PM_{2.5}$ emission remains unchanged, then the mean $PM_{2.5}$ concentration should be relatively invariant from 2015 to 2020. According to the MBC method, daily deviations of $PM_{2.5}$ concentration in each year of 2016–2020 were calculated by subtracting the April–May mean in the year from the daily observations, and the calculated daily deviations were then added to the April–May mean of 2015 to obtain $PM_{2.5}$ concentration that did not contain any effects of emission reduction. These adjusted $PM_{2.5}$ concentrations have a similar meaning to those produced by the abovementioned fixed MLR model. In all years, the MBC simulations could fully reproduce the observed CP days (Figure 6b). However, note that the simulated CP day also occurred more frequently than the observation over time (Figure 7). Although both the MLR and MBC are statistical approaches, solid and consistent results largely decreased the uncertainties.

In most of the years, the numbers of CP days simulated respectively by the MLR and MBC approaches are quite close to each other, indicating good stability of the two models (bars in Figure 7). Since the anthropogenic emission

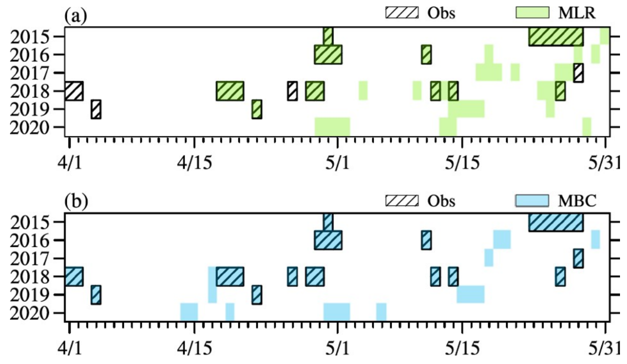

**Figure 6** Observed (black slashes) and simulated (shadings) CP days during 2015–2020. The MLR-simulated ((a), green shadings) and MBC-simulated ((b), blue shadings) $PM_{2.5}$ concentrations and observed $O_3$ concentrations are used to calculate CP days.

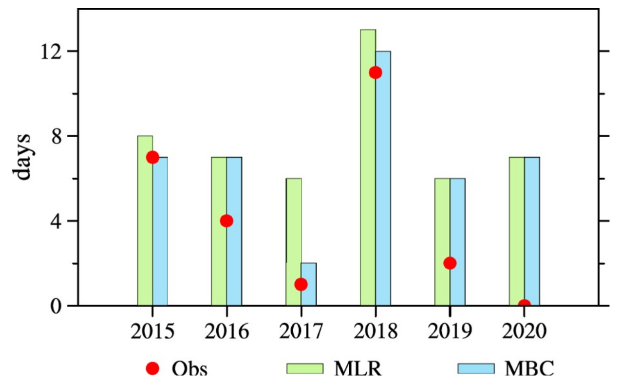

**Figure 7** Number of observed (red dots), MLR-simulated (green bar), and MBC-simulated (blue bar) CP days from 2015 to 2020. The simulated CP days were outputted by a fixed model trained by data in 2015.

was fixed at the level of 2015, the simulated number of CP days was 1–3 days (averaged of results from the two methods) more than the observed number of CP days during 2016–2018. That is, the number of CP days was reduced by 7 due to the impact of emission reduction on $PM_{2.5}$ concentration in three years. If the anthropogenic emission is maintained at the level of 2015, there would have 6 and 7 days of $PM_{2.5}$ and $O_3$ co-pollution in BTH in 2019 and 2020, respectively. In practice, the number of CP days in 2019 and 2020 was only 2 and 0 respectively, indicating that the number of $PM_{2.5}$ and $O_3$ co-pollution days was reduced by 11 due to the strict and persistent emission regulations in China. On the other hand, if the high pollutant emission level of 2015 persists, the number of CP days will remain to be 7 in the subsequent years except for 2018. This result also indicates the robust impacts of anthropogenic emissions.

## 6. Conclusions and discussions

Co-occurrence of $PM_{2.5}$ and $O_3$ pollution enhanced the complexity of pollution control and often led to superimposed hazard to local residents and the ecosystem. In this study, the characteristics of $PM_{2.5}$ and $O_3$ co-pollution in BTH and the influences of metrological conditions and emission reduction on pollution days during April–May of 2015–2020 were analyzed. On the daily time scale, the co-occurrence of the Scandinavia teleconnection pattern and the anomalous anticyclonic circulation over North China 0–2 days in advance could be responsible for approximately 70% of CP days in the BTH region. Therefore, such a configuration of atmospheric circulation could serve as a useful precursor signal for the prediction of double-high air pollution in BTH. Differently, the Rossby wave-like train was not significant during PP and OP days. At the same time, the SN pattern resulted in a hot (high temperature and strong solar radiation), moist (high humidity), and stagnant (anomalous southerlies) environment that was favorable for the increase of $PM_{2.5}$ and $O_3$ concentrations. The occurrence frequency of the SN index larger than one standard deviation was the highest in 2018 and about twice its average in other years (Figure S3). This large-scale atmospheric background tended to modulate the high concentrations of $PM_{2.5}$ and $O_3$, leading to their simultaneous occurrences. This mechanism explains why the highest number of CP days was observed in 2018. The relative humidity near the surface was much higher in 2018 than in other years (figure not shown), which also partly prevented the decrease of $PM_{2.5}$ concentration in 2018 due to emission reduction, keeping it at a relatively high level.

The level of anthropogenic emission was the main factor that influenced the changes in CP numbers in April–May from 2015 to 2020. Particularly, the on-going decline of CP days in 2019 and 2020 very likely resulted from the impact of emission reduction on $PM_{2.5}$ pollution. Total, pollution days were reduced by about 18 due to strict measurements of air pollution control during 2015–2020. Also, $PM_{2.5}$ and $O_3$ co-pollution days were not found in 2021, which further confirms the impact of emission reduction on air quality. Moreover, $O_3$ concentration exceeded the Grade II air quality standard in more than 16 days during April–May of 2019 and 2020, when temperature and solar radiation were much lower than that in summer. The serious $O_3$ pollution also needs to be addressed by rigorous and synergetic management. Although two different approaches were used in the present study to illustrate the impact of emission reduction, the statistical approaches were short of $PM_{2.5}$-$O_3$ interactions and new particle formation with changing emission intensity (Tang et al., 2021). In fact, high $O_3$ concentration and strong atmospheric oxidation could promote the formation of secondary particles and thus elevate $PM_{2.5}$ concentration (Feng et al., 2019). Meanwhile, the presence of $PM_{2.5}$ played an important role in radiative forcing and could reduce $O_3$ (Wang et al., 2019). This may cause the concentrations of $PM_{2.5}$ and $O_3$ do not reach extremely high values at the same time during CP days. Additionally, the present study emphasized the impact of emission reduction on $PM_{2.5}$ concentration but ignored those factors related to surface $O_3$ pollution that must be considered in further work. Fully coupled atmospheric chemical models provided a powerful tool to verify the conclusions of the present study based on observations and facilitate a better and deeper understanding of air pollution days in China.

**Acknowledgements** *This research was supported by the National Natural Science Foundation of China (Grant No. 42088101).*

**Open Access** This article is licensed under a Creative Commons Attribution 4.0 International License, which permits use, sharing, adaptation, distribution and reproduction in any medium or format, as long as you give appropriate credit to the original author(s) and the source, provide a link to the Creative Commons licence, and indicate if changes were made. The images or other third party material in this article are included in the article's Creative Commons licence, unless indicated otherwise in a credit line to the material. If material is not included in the article's Creative Commons licence and your intended use is not permitted by statutory regulation or exceeds the permitted use, you will need to obtain permission directly from the copyright holder. To view a copy of this licence, visit http://creativecommons.org/licenses/by/4.0/.

## Electronic supplementary material

Supplementary material, approximately 116 KB.
