# Supplementary material for: Meteorological influences on co-occurrence of O3 and PM2.5 pollution and implication for emission reductions in Beijing-Tianjin-Hebei
Source: Sci China Earth Sci. 2023 May 4:1–10. Online ahead of print. doi: 10.1007/s11430-022-1070-y (PMC10205161; doi:10.1007/s11430-022-1070-y)
Supplement: Supplementary file 1 — Supplementary material, approximately 116 KB. [file 11430_2022_1070_MOESM1_ESM.docx]

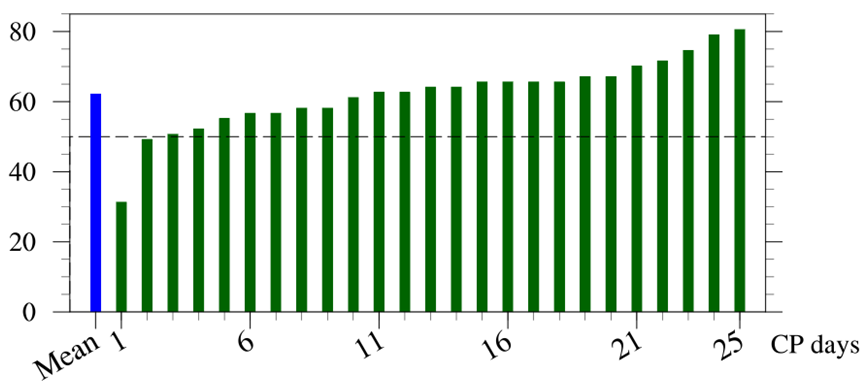


**Figure S1.** The percentage of co-occurrence of O_3_ and PM_2.5_ pollution at 67 stations during CP days. Green bars indicate the percentage of co-occurrence of O_3_ and PM_2.5_ pollution at 67 stations at each CP day. The blue bar represents the average percentage of all CP days. The horizontal dashed line indicates the percentage of 50.


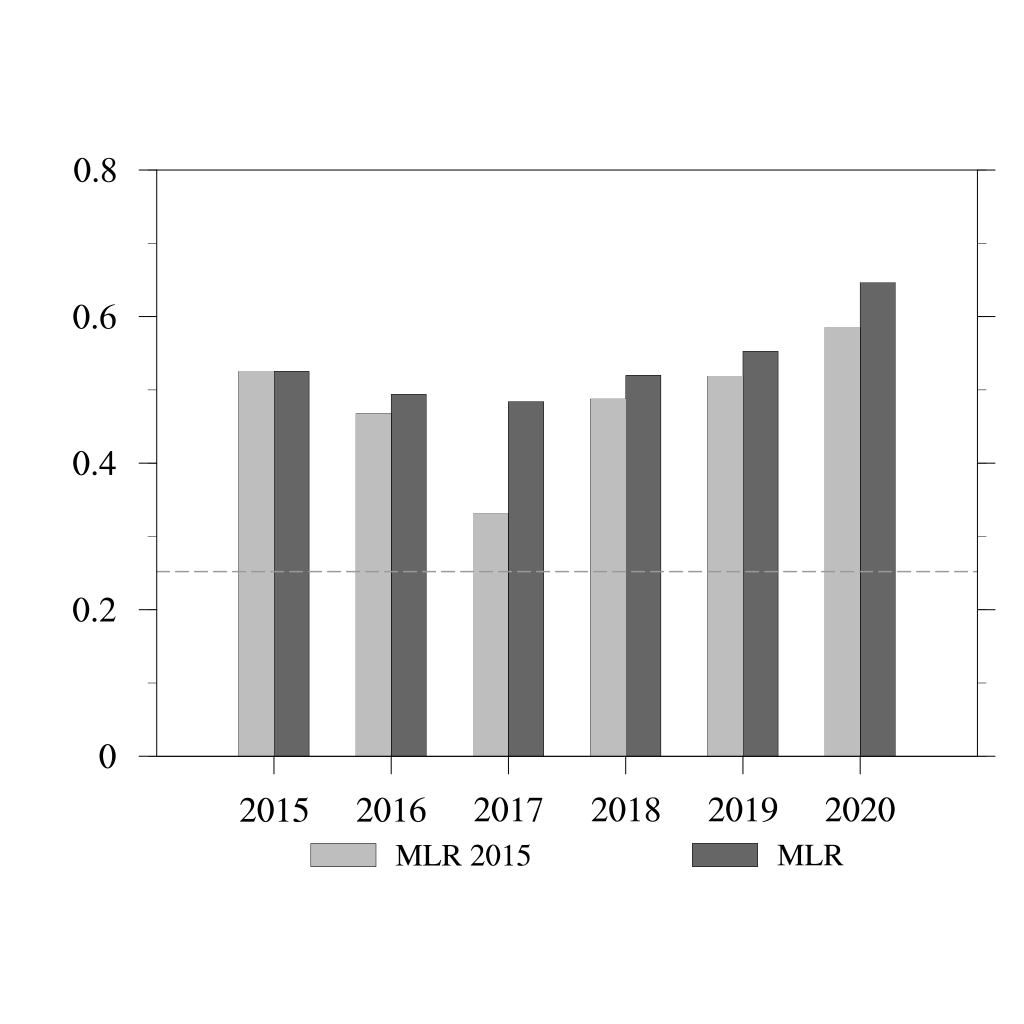


**Figure S2.** Correlation coefficients between the observed and simulated PM_2.5_ in BTH in April–May. The simulations used in black bar were fitted by the MLR model updated every year, while those associated with the grey bar were outputted by fixed model trained by data in 2015. The real-time meteorological variables were inputted into both of the update and fixed model.


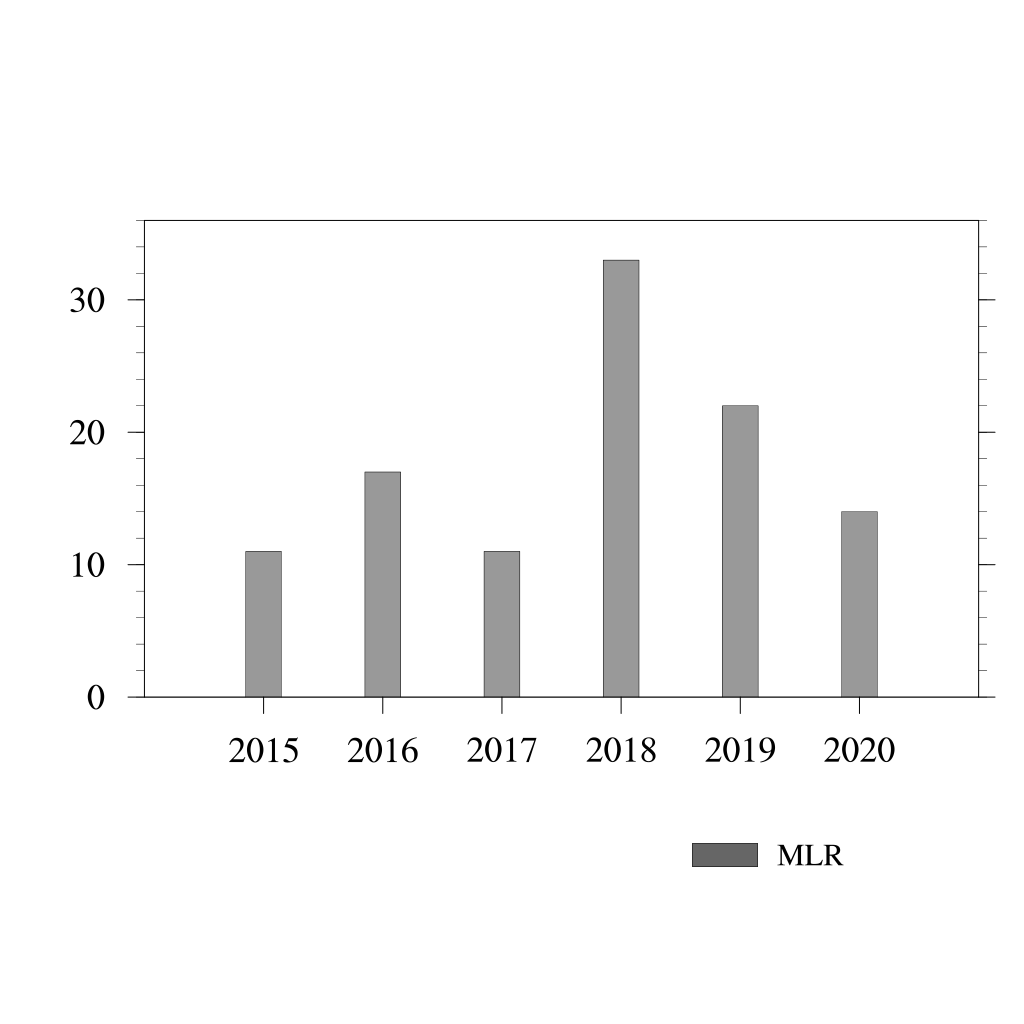


**Figure S3.** The number of days when SN index larger than one standard deviation in April and May from 2015 to 2020.
